# C-854-02. Code Blue: Training for the Big Event

**DOI:** 10.1093/jbcr/irag033.078

**Published:** 2026-04-06

**Authors:** Cheryl L Barela, Mark Romero, Stacey Richerbach, Kevin N Foster

**Affiliations:** Diane & Bruce Halle Arizona Burn Center - Valleywise Health, Phoenix, AZ; CommonSpirit Health, Phoenix, AZ; Diane & Bruce Halle Arizona Burn Center - Valleywise Health, Phoenix, AZ; Diane & Bruce Halle Arizona Burn Center - Valleywise Health, Phoenix, AZ

## Abstract

**Introduction:**

In 2024, our burn center experienced multiple rapid response events and Code Blue activations in our medical/surgical unit that resulted in poor outcomes. Post-event debriefings revealed that nurses lacked confidence in leading codes, administering emergency medications, and using code cart equipment, and that participation in the hospital’s quarterly CPR refresher program did not instill the confidence needed to adequately respond to or participate in a code. These gaps negatively affected team performance during high-acuity emergencies. To address this, a Quality Improvement initiative—the Code Cart Class—was implemented to increase familiarity with code cart contents, reinforce medication safety, and build confidence in responding to resuscitation events.

**Methods:**

The intervention combined didactic instruction, hands-on review of code cart contents, and simulation-based mock code practice. Nurses completed pre- and post-intervention surveys to assess comfort and confidence in code response, roles and responsibilities, and equipment use.

**Results:**

A total of 104 nurses completed the Code Cart Class with 65% having over 5 years of experience, 23% having 2 to 5 years of experience, and 14% having less than 2 years of nursing experience. Post-education surveys demonstrated significant improvements in locating supplies in the code cart (↑42%), confidence operating the monitor/defibrillator (↑39%), confidence responding to a code (↑38%), and comfort assisting or leading a code (↑35%). Qualitative feedback emphasized that hands-on practice and simulation reduced anxiety and enhanced preparedness.

**Conclusions:**

The Code Cart Class effectively addressed gaps in code readiness, enhancing both individual nurse confidence and team response in the burn unit. Integrating structured education with simulation provided a sustainable model for improving performance during high-acuity events and supported a stronger culture of safety.

**Applicability of Research to Practice:**

This project illustrates the impact of nurse-led education on emergency preparedness in specialized care settings.

**Funding for the Study:**

N/A.

